# Antibiotic Resistance Profiles of Escherichia coli and Salmonella spp. Isolated From Dairy Farms and Surroundings in a Rural Area of Western Anatolia, Turkey

**DOI:** 10.7759/cureus.65996

**Published:** 2024-08-02

**Authors:** Savaş Aslan, Cengiz Demir, Elçin L Kurtoğlu, Mustafa Altındiş

**Affiliations:** 1 Health Policy, Medical Laboratory Techniques Program, Şuhut Vocational School of Health Services, Afyonkarahisar Health Sciences University, Afyonkarahisar, TUR; 2 Medical Microbiology, Afyonkarahisar Health Sciences University, Afyonkarahisar, TUR; 3 Medical Genetics, Medical Laboratory Techniques Program, Şuhut Vocational School of Health Services, Afyonkarahisar Health Sciences University, Afyonkarahisar, TUR; 4 Medical Microbiology, Sakarya University, Sakarya, TUR

**Keywords:** salmonella spp, dairy farms, antibiotic resistance, pcr, escherichia coli

## Abstract

Background

Antibiotic resistance is a significant public health issue worldwide. Antibiotic-resistant zoonotic bacteria such as *Escherichia coli* (*E. coli*), *Campylobacter*, *Salmonella*, *Listeria*, *Coxiella*, and *Mycobacterium *can be particularly isolated from biofertilizers. Epidemiological studies have shown that cases of foodborne infections and intoxications are significantly related to animal-derived foods. The presence of these species in aquatic environments indicates areas or organisms contaminated with animal or human feces. Especially, the presence of *E. coli* in aquatic environments has become a serious problem worldwide. Pathogenic strains of *E. coli* cause waterborne and foodborne diseases.

Materials and methods

This study included a total of 290 samples collected from five different dairy farms between April and September 2023 which comprised 20 samples of cow manure, 20 samples of milk, three samples of dairy workers' hand washing water, five samples of soil, five samples of water, and five samples of vegetables. The samples taken from the farms were homogenized with 0.1% peptone water at a ratio of 1/10. They were then cultured on xylose lysine deoxycholate (XLD), eosin methylene blue agar (EMB), and blood agar media, and gram-negative colonies were identified using matrix-assisted laser desorption/ionization time-of-flight mass spectrometry (MALDI-TOF MS) and the VITEK2 automated system (BioMerieux Inc., Durham, NC). Amplification of the isolated DNA extracts was performed with A.B.T.™ 2X HS-PCR MasterMix (A.B.T Laboratory Industry, Arnavutköy, Turkey) in the SimpliAmp™ thermal cycler (Thermo Fischer Scientific Inc., Waltham, MA) and visualized by agarose gel electrophoresis.

Results

Among the 52 *E. coli* strains isolated in our study, the highest antibiotic sensitivity rate was observed in meropenem, while the lowest sensitivity rates were determined in cefazolin and cefuroxime. While two of the *Salmonella *spp. (n = 2) isolates were found to be resistant to tetracycline, and one was found to be resistant to penicillin and ampicillin. No resistance to trimethoprim/sulfamethoxazole was detected in either isolate. Extended-spectrum beta-lactamases (ESBLs) were detected in only four (7.7%) *E. coli* strains. While tetA, tetB, and TEM genes were seen in almost all *E. coli* strains, they were not found in *Salmonella *spp.

Conclusion

In conclusion, our study revealed the presence of antimicrobial resistance genes in *E. coli* and *Salmonella *spp. isolates collected from various farms and environmental samples, which render the antimicrobials used for disease treatment ineffective. Consequently, research should be undertaken to prevent the development of new resistance genes in our country, as creating new medications and treatment strategies for these diseases is costly and time-intensive.

## Introduction

Antibiotic resistance continues to be a global public health concern [1]. The impact of antibiotic-resistant bacteria in the etiology of infections continues to rise to concerning levels [2]. It is estimated that approximately 10 million people die each year due to antimicrobial resistance infections [3]. Drug-resistant microorganisms are being reported from all regions of the world, and their adverse effects have significantly increased in recent years [4]. The indiscriminate use of antibiotics and lack of knowledge on this subject are among the most important reasons for the proliferation, selection, and spread of antibiotic-resistant organisms [5]. Many antimicrobial agents are used in animal feed production to control diseases and are often preferred as growth-promoting factors. At the same time, they continuously spread in the human food chain, leading to serious health problems in humans and animals [6]. Cattle on dairy farms are potential sources of contamination with antibiotic-resistant *Escherichia coli* (*E. coli*) and *Salmonella* spp., which are found in cow manure and can contaminate the farm environment and farm products. Moreover, these resistance elements can cause serious human health problems by being transmitted directly to farm workers through contaminated soil, water, and milk [7].

*Escherichia coli *is recognized as a dangerous pathogen in the dairy farm sector worldwide due to the significant economic losses it causes [8]. There are various types of *E. coli*, most of which are harmless, but a few can cause serious foodborne infections in humans [9]. Farm animals, especially cattle, carry Shiga toxin-producing *E. coli* (STEC) and enterohemorrhagic *E. coli *(EHEC) asymptomatically. These pathogens are inherently zoonotic and can be transmitted from farms to humans through contaminated milk, meat, water, and direct contact with animals or their environmental equipment [10]. Dairy cows serve as reservoirs for *Salmonella *spp., which can cause human salmonellosis [11]. *Salmonella *spp. can be transmitted from infected cattle and their surroundings through feces. In recent years, the increasing resistance of *Salmonella *serotypes to commonly used antibiotics has significantly increased the cost of treatment in food animal production [12]. Animal manure contains microbial components that make it a potential source of pathogenic microorganisms for both animals and humans. Fresh farm animal manure is produced in many countries and is predominantly used as a biofertilizer in agricultural lands [3]. Various bacterial pathogens, such as *E. coli*, *Campylobacter*, *Salmonella*, *Listeria*, *Coxiella*, and *Mycobacterium*, inherently resistant to antibiotics and zoonotic, have been obtained from manure. These pathogens can enter the food chain in a way that affects consumer health when manure is used as fertilizer for crop, vegetable, and fruit production in agriculture [13].

The emergence of antibiotic-resistant bacteria and their resistance genes has become an increasingly serious problem in existing drugs. There is a lack of sufficient data on the formation of antibiotic-resistant bacteria, particularly in dairy cattle farming systems. The most serious challenge encountered in controlling and treating these pathogens is the increasing drug resistance in *Salmonella *spp. and *E. coli* strains and the development of multidrug-resistant epidemic types. Therefore, studies on the exact sources of bacterial dissemination and their genetic profiles are very important. In this study, the aim is to characterize antibiotic resistance genes in *E. coli* and *Salmonella *spp. strains isolated from dairy farms and their surroundings in the Afyonkarahisar province region of Turkey to contribute to the selection of antibiotics for empirical therapy.

This study was supported by the Afyonkarahisar Health Sciences University Scientific Research Projects Coordination Unit (project number: 19.SHMYO.001).

## Materials and methods

Sample collection

From each dairy farm, a total of 58 samples were collected, comprising 20 samples of cow manure, 20 samples of milk, three samples of handwashing water from milkers, five soil samples, five water samples, and five vegetable samples (such as spinach, green pepper, and tomato), resulting in a total of 290 samples collected from five different farms. Cow fecal samples were collected immediately after defecation while handwashing samples from dairy farm workers, soil samples, and vegetable samples were collected when sampling vegetables like spinach, green pepper, and tomato.

Sample processing

Two different types of samples, solid (cow feces, soil, and vegetables) and liquid (milk, handwashing water from milkers, and water), were measured in grams and milliliters, respectively. For cow feces and soil samples, 10 g of each sample was taken and homogenized in 90 ml of 0.1% peptone water. Collected vegetable samples were divided into small pieces with a sterile knife and thoroughly mixed to obtain a homogeneous mixture. Twenty-five grams of the mixed diced vegetables were homogenized in 225 ml of 0.1% peptone water. For preparing liquid samples, 10 ml of the sample was mixed with 90 ml of diluent for the initial dilution. Finally, 10-fold serial dilutions were made from all initial dilutions for bacterial enumeration.

Identification of bacterial strains and antibiotic susceptibility testing

Samples were cultured on blood agar, xylose lysine deoxycholate (XLD) agar, and eosin methylene blue (EMB) agar plates. The plates were then incubated at 37°C for 24 hours. Following incubation, colonies identified as Gram-negative were subjected to species-level identification using the matrix-assisted laser desorption/ionization time-of-flight mass spectrometry (MALDI-TOF MS) (Bruker Daltonics, Bruker Microflex LT, Bremen, Germany) and VITEK 2 automated systems (BioMerieux Inc., Durham, NC). Until susceptibility and molecular tests were conducted, strains were suspended in 2 ml sterile Eppendorf tubes containing tryptic soy broth supplemented with 15% glycerol and stored at -80°C. The VITEK 2 automated system was utilized to determine the antibiotic susceptibility of the bacterial strains included in the study. Standard strains of *E. coli* American Type Culture Collection (ATCC) 25922 and *Salmonella typhimurium* ATCC 14028 were used for all susceptibility tests.

Bacterial DNA isolation

The DNA extraction was performed using the boiling method. For this purpose, three to five colonies from fresh passages on sheep blood agar were aseptically picked with a sterile loop. These colonies were then suspended in 300 µl of distilled water in labeled sterile Eppendorf tubes, ensuring complete dissolution. Subsequently, the tubes were placed in a water bath filled with water and foam spacers at 95°C for 20 minutes. After incubation, the Eppendorf tubes were centrifuged at 14,000 rpm for five minutes at room temperature. Following centrifugation, 100 µl of the supernatant was used as the template DNA for amplification.

DNA amplification

The DNA samples were amplified using specific primers and A.B.T.™ 2X HS-PCR MasterMix (with BlueDye) (P02-02-01, Turkey) from A.B.T Laboratory Industry, Arnavutköy, Turkey, in the SimpliAmp™ thermal cycler (Thermo Fischer Scientific Inc., Waltham, MA). The thermal cycling conditions were set as follows: initial denaturation at 95°C for five minutes, followed by 40 cycles of denaturation at 95°C for 15 seconds, annealing at 60°C for 30 seconds, extension at 72°C for 30 seconds, and a final extension step at 72°C for five minutes. After amplification, PCR products were visualized using 2% agarose gel electrophoresis. The primer pairs used in resistance gene detection in the obtained isolates ranged between 169 base pair (bp) and 200 bp (Table 1).

**Table 1:** Primers used for the detection of gene regions

Genes	Forward (F)/reverse (R)	Primers	Base pair (bp)	References
ereA	F-Primary	5’- ACCGCACGTTGATATGTTGA -3’	182 bp	Volokhov et al., 2003 [14]
	R- Primary	5’- CAAATCGCTGTTGACGTGTT -3’		
tetA	F- Primary	5’- AATTTCCTGACGGGCTGTTT -3’	200 bp	Wu et al., 2024 [15]
	R- Primary	5’- TGTCCGACAAGTTGCATGAT -3’		
tetB	F- Primary	5’- TTCATTAGCGGGTCTTGGTC -3’	176 bp	Parson et al., 2024 [16]
	R- Primary	5’- CCACCACCAGCCAATAAAAT -3’		
SHV	F- Primary	5’- CTTTCCCATGATGAGCACCT -3’	193 bp	Parson et al., 2024 [16]
	R- Primary	5’- CGCTGTTATCGCTCATGGTA -3’		
TEM	F- Primary	5’- TTTGCCTTCCTGTTTTTGCT -3’	169 bp	Alawi et al., 2024 [17]
	R- Primary	5’- ATAATACCGCGCCACATAGC -3’		
OXA	F- Primary	5’- GGATAAAACCCCCAAAGGAA -3’	169 bp	Parson et al., 2024 [16]
	R- Primary	5’- AAGCTACTTTCGAGCCATGC -3’		
CTX-M	F- Primary	5’- GGTGATGAACGCTTTCCAAT -3’	199 bp	Ellem et al., 2011 [18]
	R- Primary	5’- TCAATTTGTTCATGGCGGTA -3’		

## Results

In this study, a total of 290 samples collected from five different farms were included. Out of the 290 samples consisting of feces, milk, vegetables, soil, animal irrigation/vegetable irrigation water, and handwashing water, a total of 183 microorganisms were isolated. These 183 microorganisms comprised 44 different species in total. The most frequently isolated microorganisms in our study were *E. coli* (28.4%), *Enterobacter cloacae* (15.8%), *Klebsiella pneumoniae* (4.4%), and *Klebsiella aerogenes* (4.4%) (Table 2).

**Table 2:** Distribution of isolated microorganisms at the species level

Microorganism	Number	%
Acinetobacter baumannii	3	1.6
Acinetobacter baylyi	2	1.1
Acinetobacter calcoaceticus	4	2.2
Acinetobacter calcoaceticus	1	0.5
Acinetobacter courvalinii	3	1.6
Acinetobacter dijkshoorniae	1	0.5
Acinetobacter johnsonii	1	0.5
Acinetobacter pittii	5	2.7
Acinetobacter vivianii	1	0.5
Buttiauxella warmboldiae	1	0.5
Citrobacter braakii	4	2.2
Citrobacter freundii	4	2.2
Comamonas kerstersii	2	1.1
Enterobacter bugandensis	4	2.2
Enterobacter cloacae	29	15.8
Enterobacter ludwigii	1	0.5
Enterococcus faecalis	2	1.1
Escherichia coli	52	28.4
*Exiguobacterium *spp*.*	1	0.5
Klebsiella aerogenes	8	4.4
Klebsiella oxytoca	4	2.2
Klebsiella pneumoniae	8	4.4
Klebsiella variicola	4	2.2
Kosakonia cowanii	1	0.5
Lactobacillus curvatus	1	0.5
Lactobacillus rhamnosus	1	0.5
Lactococcus lactis	1	0.5
Mixta calida	1	0.5
Proteus mirabilis	2	1.1
Proteus vulgaris	1	0.5
Pseudomonas aeruginosa	3	1.6
Pseudomonas cichorii	1	0.5
Pseudomonas koreensis	2	1.1
Pseudomonas monteilii	1	0.5
Pseudomonas otitidis	1	0.5
Pseudomonas plecoglossicida	1	0.5
Pseudomonas protegens	1	0,5
Pseudomonas putida	6	3.3
Pseudomonas putida	4	2.2
Pseudomonas thivervalensis	1	0.5
Raoultella ornithinolytica	1	0.5
*Salmonella *spp*.*	2	1.1
Serratia liquefaciens	2	1.1
Serratia mercescens	4	2.2
Total	183	100

Identification revealed that 52 isolates were *E. coli*, while two were identified as *Salmonella *spp. Antibiotic susceptibility testing was conducted for *E. coli* strains. Among the 52 *E. coli* strains, the highest antibiotic susceptibility rate was observed for meropenem, while the lowest susceptibility rate was detected for cefazolin and cefuroxime antibiotics (Table 3). While two of the *Salmonella *spp. (n = 2) isolates were found to be resistant to tetracycline, and one was found to be resistant to penicillin and ampicillin. No resistance to trimethoprim/sulfamethoxazole was detected in either isolate. Extended-spectrum beta-lactamases (ESBLs) were detected in only four (7.7%) *E. coli *strains.

**Table 3:** Antibiotic susceptibility profiles of Escherichia coli isolates

Antibiotics	Escherichia coli	
	Sensitive (%)	Resistant (%)
Amikacin	94.2	5.8
Amoxicillin/clavulanic acid	75	25
Ampicillin	69.2	30.8
Cefazolin	0	100
Cefepime	94.2	5.8
Cefoxitin	94.2	5.8
Ceftazidime	92.3	7.7
Ceftriaxone	92.3	7.7
Cefuroxime	0	100
Ciprofloxacin	80.8	19.2
Colistin	94.2	5.8
Ertapenem	96.2	3.8
Gentamicin	92.3	7.7
Meropenem	100	0
Piperacillin/tazobactam	94.2	5.8
Tigecycline	96.2	3.8
Trimethoprim/sulfamethoxazole	82.7	17.3

In this study, the molecular analysis of seven different gene regions responsible for antibiotic resistance was conducted. The presence of ereA, tetA, tetB, SHV, TEM, OXA, and CTX-M genes was investigated, and the gel electrophoresis images of the targeted gene regions were evaluated using markers ranging from 25 bp to 700 bp in size.

The tetA gene region (200 bp)

**Figure 1:**
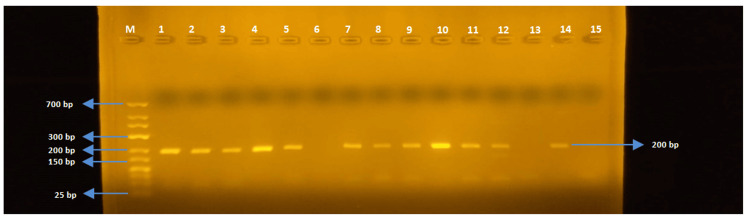
Samples one to 15 for tetA

**Figure 2:**
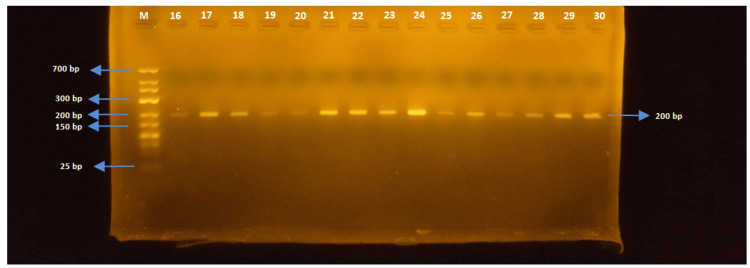
Samples 16-30 for tetA

**Figure 3:**
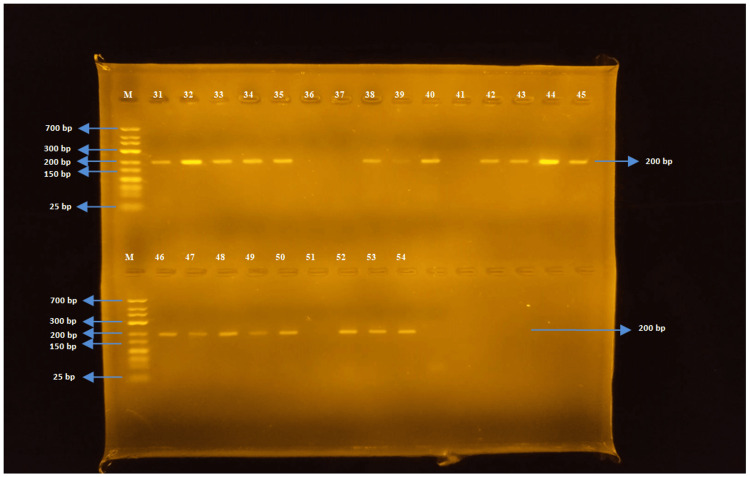
Samples 31-54 for tetA

The PCR was performed on *E. coli *and *Salmonella *spp strains with tetA-F and tetA-R primers. While the tetA gene was detected in 47 of 52 *E. coli *strains, the tetA gene region could not be detected in any of the *Salmonella *spp. strains (Figures 1-3).

The tetB gene region (176 bp)

**Figure 4:**
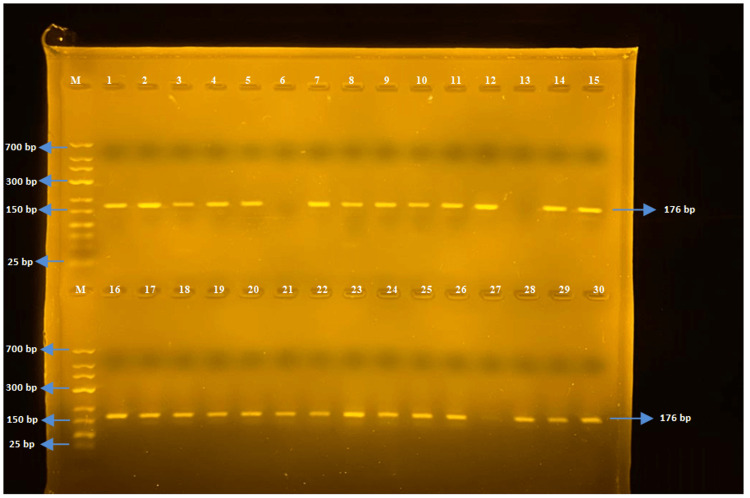
Samples one to 30 for tetB

**Figure 5:**
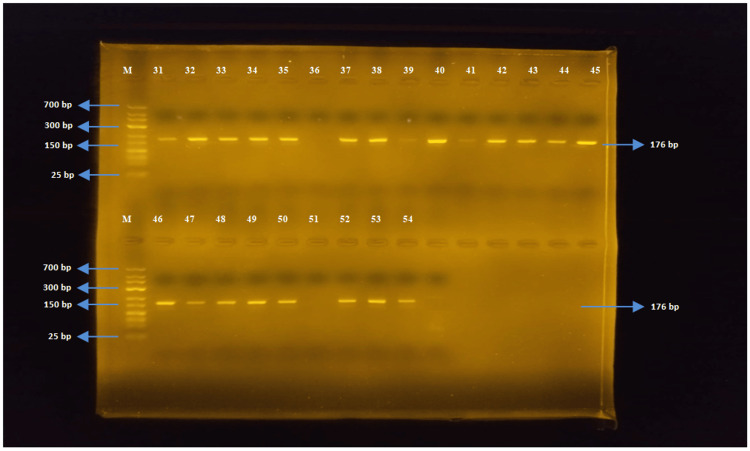
Samples 31-54 for tetB

The PCR was performed using tetB-F and tetB-R primers for *E. coli *and *Salmonella* spp. strains. The tetB gene was detected in 50 out of 52 *E. coli* strains, while none of the *Salmonella *spp. strains detected the tetB gene region (Figures 4, 5).

The TEM gene region (169 bp)

**Figure 6:**
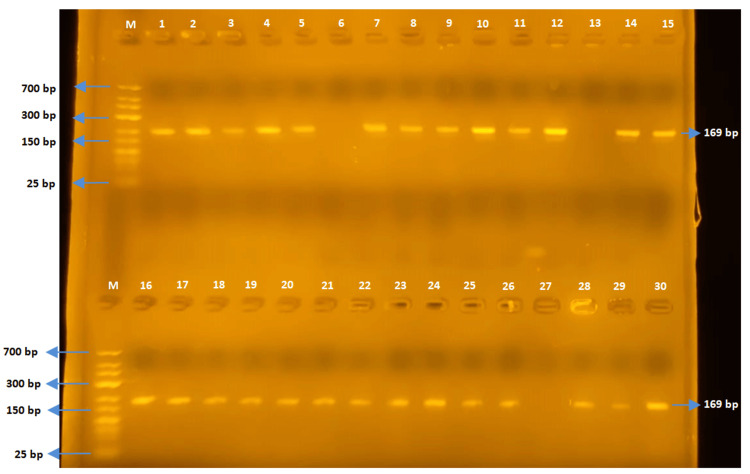
Samples one to 30 for TEM

**Figure 7:**
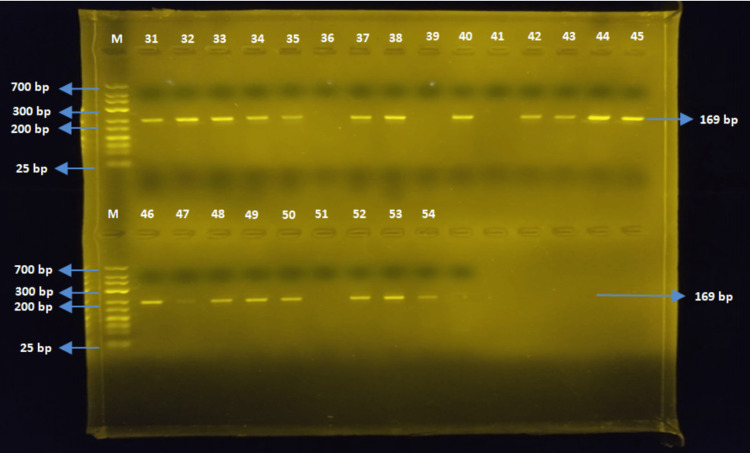
Samples 31-54 for TEM

The PCR was conducted using TEM-F and TEM-R primers for *E. coli* and *Salmonella *spp. strains. The TEM gene was detected in 48 out of 52 *E. coli* strains, while none of the *Salmonella* spp. strains detected the TEM gene region (Figures 6, 7).

Other gene regions

In the PCR study, OXA, CTX-M, SHV, ereA gene regions could not be detected in any of the 52 *E. coli* and two *Salmonella* spp. strains.

## Discussion

Antimicrobial resistance poses a serious global public health concern [19]. Inappropriate use of antibiotics by humans, factories, and farms, poor hygiene, and the inability to prevent infections in healthcare facilities are considered significant contributors to the emergence and spread of antibiotic-resistant bacteria [20]. Extended-spectrum beta-lactamases are enzymes that confer resistance to most β-lactam antibiotics, including penicillins, cephalosporins, and the monobactam aztreonam. Infections with ESBL-producing organisms have been associated with poor outcomes [21]. *Escherichia col*i is a notable example of microorganisms that develop antibiotic resistance, particularly multidrug resistance (MDR), and can cause life-threatening infections by producing ESBLs [22]. *Escherichia coli* is a facultative member of the flora predominant in the gastrointestinal system of both humans and animals [23]. Prolonged exposure of *E. coli* and *Salmonella* spp. to antibiotics contributes to the development of antibiotic resistance. Therefore, antibiotic-resistant bacteria, including *E. coli* and *Salmonella *spp. in animals, can serve as significant reservoirs for human colonization and infection. Studies have shown that antibiotic-resistant bacteria can spread from the environment to humans through direct or indirect contact (e.g., consuming contaminated food and water) [24]. Therefore, evaluating the prevalence of antibiotic-resistant *E. coli* and *Salmonella* spp. from different sources is of critical importance for establishing guidelines in veterinary and human health services. Particularly in developed countries, bacterial resistance monitoring programs are regularly conducted, and the results regarding the resistance status of indicator, pathogenic, and zoonotic bacteria to antibiotics are periodically published [25]. As *E. coli* is a natural member of human and animal intestinal flora, it can frequently be exposed to antibiotics used for various purposes in animals, thereby easily developing various resistance mechanisms. Another characteristic of *E. coli* is its ability to transfer resistance genes to pathogenic and zoonotic bacteria, thus being considered an indicator bacterium in monitoring antibiotic resistance [26]. Therefore, it is crucial to examine not only pathogenic bacteria but also normal flora members like *E. coli* isolates in monitoring antibiotic resistance.

Bacteria within the *Enterobacterales *family have a wide range of hosts, including plants, insects, animals, and humans. Some species of this family are found in the normal intestinal flora of humans and animals [27]. These bacteria are not only pathogens or members of the intestinal flora but are also abundantly found in almost every moist environment, particularly in soil, water, and household settings [28]. In their study, Naderi et al. (2024) [29] analyzed the prevalence of antibiotic resistance genes, phenotypic resistance, and all percentage rates associated with phylogenetic groups among 51 virulence gene-positive *E. coli* isolates from 36 healthy and 15 diarrheal calves. This study detected 9.8% tetA, 5.9% tetB, 3.9% TEM, and 3.9% bla SHV genes. In their study, Jia et al. (2022) [30] investigated multiple resistance genes found in ultra-broad-spectrum beta-lactamases (ESBL) and AmpC enzymes. In this study, bla CTX-M, bla TEM, and bla SHV were detected at a rate of 29%, 29%, and 9.5%, respectively. In their study of molecular characterization of *E. coli* strains by Gautam et al. (2019) [31], it was shown that ESBL and AmpC enzymes are commonly found in *E. coli* of animal origin, the frequency of beta-lactamase antimicrobial drugs has increased, and bla CTX M and bla TEM types are currently more widely distributed genotypes. In a study by Geser et al. (2012) [32] conducted in Switzerland, they found ESBL-producing *Enterobacterales *isolates in 25.3% of animal fecal samples they collected. When the studies conducted in Turkey were examined, the ESBL-producing *E. coli* strain was not found in adult cattle in the study conducted by Aksoy et al. [33] and Buyuknal et al. [34]. In the survey conducted in Hatay, the prevalence was found to be 8.3% in adult cattle [35]. In a study conducted on fecal samples collected from the Burdur region, the rate of ESBL-producing *E. coli* was found to be 15.5% [36]. In our study, ESBL was detected in 4 (7.7%) of the isolated *E. coli* strains. It is observed that the rate of ESBL-producing strains in our study is consistent with other studies conducted in our country, which are important data in observing resistance development in bacteria. Beta-lactams are a group of antibiotics that inhibit cell wall synthesis at various stages. They constitute more than 50% of the antibiotics consumed worldwide [37]. In our study, the susceptibility rates of beta-lactam antibiotics in isolated *E. coli* strains were investigated. Resistance against ampicillin was detected at a rate of 30.8%. Many studies have shown that high levels of resistance to beta-lactam antibiotics, which are commonly used in clinical practice, are found in enteric bacteria isolated from various environments [38]. Resistance to this group of antibiotics is generally found in transferable resistance plasmids (R-plasmids), which leads to the rapid spread of resistance among the same or different species of microorganisms [39]. In a study conducted in our country, the blaTEM gene, encoding TEM-type beta-lactamase, was investigated in 96 ampicillin-resistant strains. As a result of the study, two strains were found to be positive for the blaTEM gene [40]. Beta-lactamase enzymes can be chromosomal or plasmid-mediated. Although plasmid-mediated beta-lactamases are commonly found in enteric bacteria, the most common type is reported to be TEM-1 among TEM-type beta-lactamases [41]. In a study by Yıldırım et al. (2018) [42], the TEM gene was investigated, including 44 samples. They identified the TEM gene in 33 (91.66%) of the 36 ESBL-producing *E. coli* isolates. The higher prevalence of this gene could be attributed to more frequent and over-the-counter use of antimicrobials in our country, as well as differences in antimicrobial prescribing practices and hygiene control measures. In the same study, the SHV gene region was also investigated but was not detected in any strain. While different rates of the TEM gene were found in studies conducted in our country, in our study, neither the TEM nor the SHV gene was detected in any of the 54 strains. Hemeg et al. (2018) [43] examined 120 *E. coli* isolates and detected the blaTEM ampicillin resistance gene in all of these isolates using PCR, highlighting the need to not only focus on patients in the classification of hospital and community-acquired infections. Van et al. (2008) [44] isolated 38 *E. coli* strains in their study conducted in Vietnam and used PCR analysis to detect the presence of some important antimicrobial resistance genes. They found the blaTEM gene in 84.2% of these *E. coli* isolates [44].

Tetracycline antibiotics are among the most widely used antibiotics in animal husbandry and agriculture, in addition to their use in the treatment of human infections. Tetracyclines constitute about 3% of the world's antibiotic production. It has been shown that members of the *Enterobacterales *family have at least 15 different genes that confer resistance to tetracyclines, and these genes may be plasmid, transposon, or chromosomal in origin [45]. In a study by Sevim et al. (2016) [40], 24 tetracycline-resistant isolates were examined for the presence of tetA, tetB, and tetC genes, and it was reported that the tetB gene was widespread among the isolates, with eight isolates containing the tetB gene and two containing the tetA gene. In our study, the tetA gene was detected in 47 (90.4%) of the isolated *E. coli* strains, and the tetB gene was detected in 50 (96.2%) of them. In a study conducted in our country using PCR with CTX-M universal primers, it was reported that all isolates (44 isolates) examined carried the CTX-M gene. Subsequently, in PCR using CTX-M group 1 primers, it was reported that all isolates carried CTX-M genes belonging to Group 1 [42]. Kürekci et al. (2019) [46] isolated 52 *E. coli* strains from various markets and butchers in Hatay and identified the CTX-M gene in 31 (62.3%) of them and the TEM gene in 19 (36.5%) of them. They reported that animal-derived foods significantly risk extraintestinal *E. coli* infections producing ESBLs. Nevertheless, they suggested that analyzing clinically ESBL-producing *E. coli* isolates together with those isolated from animal-derived foods would help better understand their potential source in Turkey. In our study, PCR was performed using CTX-M-F and CTX-M-R primers for *E. coli* and *Salmonella *spp. strains. The CTX-M gene region was not detected in any of the *E. coli* strains or *Salmonella *spp. strains. In a study by Dehdashti et al. (2019) [47], none of the 49 isolated *E. coli* isolates were found to carry CTX-M antimicrobial resistance genes similar to our study. Pehlivanlar Onen et al. (2015) [48] collected 100 chicken and 100 meat samples from various markets and butchers and isolated beta-lactamase-producing *E. coli* from 81 chicken samples and seven meat samples. They detected the blaCTX-M gene in 60 of the chicken isolates and 19 of the meat isolates, and the blaTEM gene in 19 of the chicken isolates and two of the meat isolates using PCR, reporting that retail-sold meat, especially chickens, was highly contaminated with ESBL-producing *E. coli*. Macrolides are used to treat infections because they are reliable and have good efficacy. Macrolide resistance can have significant consequences for public health. In a study by Phuc Nguyen et al. (2009) [49], the ereA gene was not detected in any of the 190 *E. coli* isolates. In a study by Dehkordi et al. (2014) [50], the ereA gene was detected in nine (18%) of the 50 isolated *E. coli* isolates. In a study conducted in our country by Keskin (2019) [51], the ereA gene was not detected. The ereA gene region was also not found in our study, which examined 54 strains.

The relatively small sample size included in this study limits the ability of the obtained data to represent the general population. Additionally, our analysis is based on data collected over a specific period. Therefore, in such cases, causal relationships cannot be fully determined. Studies that collect data repeatedly from the same populations over time can provide a better understanding of causal relationships. To this end, using larger and more diverse samples can increase the generalizability of the findings to a broader population.

## Conclusions

In conclusion, according to the data obtained in our study, antimicrobial resistance genes that render antimicrobials used to treat diseases ineffective were detected in *E. coli *and *Salmonella *spp*.* isolates isolated from various farms and environmental samples investigated in our study. Therefore, studies should be conducted to prevent the emergence of new resistance genes in our country, as developing new drugs and treatment methods for these diseases is expensive and time-consuming. Since various diseases occur due to food contamination with *E. coli *and *Salmonella *spp*.*, hygiene rules must be followed in the slaughter, storage, and transportation of these foods and on the farms where these foods are obtained. The results of this study have demonstrated that antimicrobial resistance genes are quite common in animal-derived foods consumed by humans using molecular methods and have shown that there is a potential reservoir for the transmission of antimicrobial resistance from animals to humans. This study will contribute to the measures to be taken for public health in our country and will provide a knowledge base for more advanced studies on this issue.
